# Facile metagrating holograms with broadband and extreme angle tolerance

**DOI:** 10.1038/s41377-018-0075-0

**Published:** 2018-10-17

**Authors:** Zi-Lan Deng, Junhong Deng, Xin Zhuang, Shuai Wang, Tan Shi, Guo Ping Wang, Yao Wang, Jian Xu, Yaoyu Cao, Xiaolei Wang, Xing Cheng, Guixin Li, Xiangping Li

**Affiliations:** 1 Guangdong Provincial Key Laboratory of Optical Fiber Sensing and Communications, Institute of Photonics Technology, Jinan University, 510632 Guangzhou, China; 2 Department of Materials Science and Engineering, Southern University of Science and Technology, 518055 Shenzhen, China; 3 Shenzhen Institute for Quantum Science and Engineering, Southern University of Science and Technology, 518055 Shenzhen, China; 4 College of Electronic Science and Technology, Shenzhen University, 518060 Shenzhen, China; 5 Materials Characterization and Preparation Center, Southern University of Science and Technology, 518055 Shenzhen, China; 6 Institute of Modern Optics, Key Laboratory of Optical Information Science and Technology, Nankai University, 300350 Tianjin, China

## Abstract

The emerging meta-holograms rely on arrays of intractable meta-atoms with various geometries and sizes for customized phase profiles that can precisely modulate the phase of a wavefront at an optimal incident angle for given wavelengths. The stringent and band-limited angle tolerance remains a fundamental obstacle for their practical application, in addition to high fabrication precision demands. Utilizing a different design principle, we determined that facile metagrating holograms based on extraordinary optical diffraction can allow the molding of arbitrary wavefronts with extreme angle tolerances (near-grazing incidence) in the visible–near-infrared regime. By modulating the displacements between uniformly sized meta-atoms rather than the geometrical parameters, the metagratings produce a robust detour phase profile that is irrespective of the wavelength or incident angle. The demonstration of high-fidelity meta-holograms and in-site polarization multiplexing significantly simplifies the metasurface design and lowers the fabrication demand, thereby opening new routes for flat optics with high performances and improved practicality.

## Metasurfaces: Wide-Angle Holograms

The use of plasmonic metasurfaces has enabled the realization of broadband holograms that can operate at extreme angles approaching grazing incidence. Zi-Lan Deng and coworkers from Jinan University, Southern University of Science and Technology, Shenzhen University and Nankai University fabricated the holograms from an array of closely spaced, identical silver nanorods (90nm wide, 200nm long) on top of a silicon substrate coated with a thin layer of silver and then a layer of SiO2. The phenomenon of extraordinary optical diffraction allows highly efficient diffraction at very large angles and customizing the spacing of the nanorods allows the desired phase profile to be programmed, into the surface. The design, which operates in the visible and near-infrared range, may prove useful for various holographic applications including data encryption, anti-counterfeiting and 3D displays.

## Introduction

Optical metasurfaces composed of arrays of structured meta-atoms have attracted tremendous interest due to their versatile capabilities for tailoring the wavefront of light by locally manipulating the phase, amplitude, and polarization^1–9^. Superior to their traditional bulky counterparts, metasurface optical elements can achieve high efficiencies^10,11^, high fidelities^12,13^, multiple functionalities^14–17^, achromatic properties^18–21^, and full-color performances^22–25^ in an ultrathin interface, which can be readily integrated into miniaturized photonic devices. As such, phase-gradient metasurfaces can be generated with a subwavelength thickness by altering the shape, size, or orientation of the meta-atoms to realize various ultrathin flat optical elements, including metalens^26–31^, meta-polarizers^32–38^, and meta-holograms^39–45^. The underlying physics of a phase -gradient metasurface relies on engineering the local phase to impart the necessary transverse momenta to the impinging wavefront and route the beam toward the desired direction^46^. However, this method heavily relies on a library of intractable meta-atoms with various geometries and sizes to produce customized phase profiles, which is challenging due to the requirement of the precise lithography technique and the limited discrete phase levels. Most importantly, the phase manipulation mechanism for phase-gradient metasurfaces only works precisely at an optimal incidence angle for given wavelengths. Meta-atoms with various complex geometries produce inaccurate phase responses for distorted transverse momenta once they operate at wavelengths and incident angles that deviate from the optimal design. Thus, the metasurface device fundamentally suffers from a narrow incident angle range in a limited bandwidth.

In this study, we utilized an alternative design principle and revealed that the metagratings based on extraordinary optical diffraction (EOD) can achieve arbitrary wavefront shaping with extreme angle tolerance in a broadband spectral range. The meta-atoms in the EOD metagratings were formed by uniformly sized plasmonic nanorods, and the local periodicity between the meta-atoms determines a discrete set of diffraction channels. Based on EOD^17,19,47^, an architecture, in which plasmonic nanorods were placed on top of a dielectric spacer with a metallic background, was employed to funnel the impinging light into the desired diffraction channel with near-unity efficiency. By further continuously displacing the nanorod within each unit cell according to the strategy of a detour phase^48^, customized phase profiles for molding arbitrary wavefronts of light can be generated. Because the phase modulation rule for a EOD metagrating is intrinsically independent of the incident angles and wavelengths, the wavefront shaping capabilities are robust for a broad bandwidth and for an extremely large range of incident angles (close to the grazing incidence). Our proposed EOD metagrating does not rely on complicated and asymmetric meta-atom inclusions^49–54^ and thus significantly simplifies the metasurface design procedures and lowers the fabrication demand, which is highly desirable for various holographic applications, including high-fidelity three-dimensional displays, data encryption, and anti-counterfeiting.

## Results

**Fig. 1:**
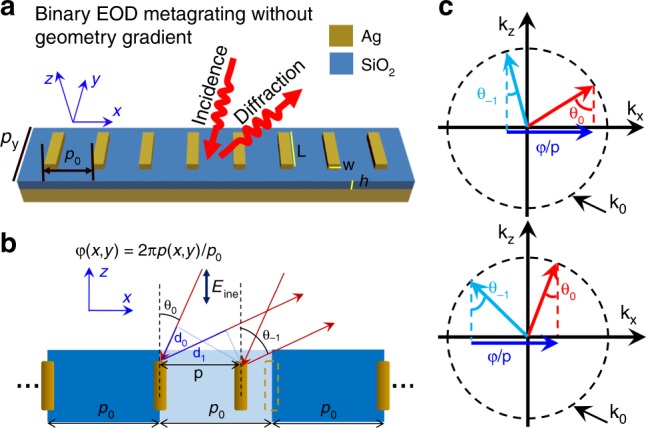
Concept of the EOD metagrating and its phase modulation rule. **a** Schematic illustration of the EOD metagrating with a periodicity of p_0_ and p_y_ in the x and y directions, respectively, composed of uniformly sized plasmonic nanorods with a width *w* and a length *L*, a dielectric spacer with thickness *h* and a metallic background. The binary metagrating supports the near-unity diffraction efficiency without any geometry gradient. **b** The phase modulation rule of metagrating. By shifting the nanorod from its original periodic lattice, a phase retardation, φ, that is proportional to the displacement, *p*, is produced in the -first diffraction direction of θ_-1_, which is dispersionless against both the incident angle, θ_0_, and the wavelength, λ. **c** The momentum relation between the zeroth and -first diffracted light at large and small incident angles, illustrating that the imparted transverse momentum, φ/p, by the meta-atoms is independent of the incident angle. θ_0_ and θ_-1_ represent the incident and diffracting angle, respectively. The dashed circles with radius k_0_ = 2π/λ represent the wave vector of light

Figure 1a shows an illustration of the proposed EOD metagrating composed of periodic arrays of identical plasmonic nanorods with width *w* and length *L*, which are placed above a dielectric spacer of thickness *h* and a metallic background. The facile binary EOD metagrating achieves near-unity efficiency light steering without the necessity of spatially varying meta-atoms to mimic a blazed grating (Supplementary Fig. S1). The EOD metagrating works in specific diffractive regimes, where only zeroth and −first (or first) diffraction orders are allowed to propagate in free space. Those EOD regimes can be determined by the following conditions,

1a $$\frac{{2\pi }}{{p_0}} - k_0 \, < \, k_x \, < \, \frac{{4\pi }}{{p_0}} - k_0\;{{\& }}\;k_0 - \frac{{2\pi }}{{p_0}} < \, k_x \, < \, k_0\;\left( {{{\rm for}}\;k_x \, > \, 0} \right)$$

1b $${k_0 - \frac{{2\pi }}{{p_0}}\, > \, k_x,\ > \, k_0 - \frac{{4\pi }}{{p_0}}\;{\mathrm{\& }}\;\frac{{2\pi }}{{p_0}} - k_0 \, > \, k_x \, > \, - k_0\;\left( {{\rm{for}}\;k_x \, < \, 0} \right)}$$

where *k*_*x=*_*k*_*0*_sinθ_0_ and k_0_ = 2π/λ are the parallel and overall wave vectors of the incident light, respectively, and θ_0_ and λ are the incident angle and wavelength, respectively. These conditions can be illustrated by the diffraction order chart shown in Supplementary Fig. S1c, in which the green patches surrounded by the zeroth, ± first, ± second Wood’s anomalies (WAs) indicate the incident angle and wavelength range determined by Eqs. (1a) and (1b). The near-unity diffraction efficiency and suppression of the zero-order diffraction can be achieved when the localized plasmonic resonance in each unit cell is tuned to be within the EOD regime^47,53^. For arbitrary wavefront shaping, one can exploit the displacement of the meta-atoms shifted from their original sites in a periodic lattice (Fig. 1b), which produces a detour phase that is proportional to displacement. In contrast to the reported lattice resonance effects^55^ by varying the distances between multiple elements in a supercell to modulate the phase, our proposed metagrating simply exploits the displacement between adjacent unit cells that each contain a single meta-atom. For a periodic metagrating with periodicity *p*_*0*_ in the *x*-direction, the momentum conservation gives that

2 $$k_0\sin \theta _0 - 2\pi /p_0 = k_0{\rm{sin}}\left( { - \theta _{ - 1}} \right)$$

where *θ*_*0*_ and *θ*_*−1*_ are the incident angles and the −first diffraction angle of light, respectively. Here, the parallel components of the incidence and diffraction wave vectors are $k_{0x} = k_0\sin \theta _0$ and $k_{ - 1x} = k_0\sin \left( { - \theta _{ - 1}} \right)$, respectively, which have opposite signs. When the meta-atom is positioned with a shifted displacement, *p*, the phase retardation of the diffracted light due to the optical path difference can be expressed as (Fig. 1b)

3 $$\phi \left( {\,x,y} \right) = k_0\left( {d_0 + d_1} \right) = k_0p\left( {\sin \theta _0 + \sin \theta _{ - 1}} \right)$$

where *d*_*0*_ = *p*sin*θ*_*0*_ and *d*_*1*_ = *p*sin*θ*_*−1*_ represent optical path differences on the incident and diffraction side, respectively. Combining Eqs. (2) and (3), we can obtain

4 $$\phi \left( {\;x,y} \right) = 2\pi p\left( {x,y} \right)/p_0$$

Thus, the detour phase is only determined by the displacement *p(x,y)* of the meta-atoms, regardless of the incident angle, *θ*_*0*_, or the wavelength, λ. Therefore, the angles of incidence and diffraction for the EOD metagratings are firmly locked and dictated by periodicity. In addition, we can modulate the phase retardation of each meta-atom from -π to π by continuously adjusting its displacement in each unit cell. As shown in Fig. 1c, for both large and small incident angles, the transverse momentum difference imparted by the EOD metagrating between the incident light and its −first diffraction order maintains the same value, φ/p, which is determined by phase retardation, φ, induced by the displaced meta-atoms. As a result, the phase modulation rule for the metagrating promises an extreme angle tolerance for the modulated wavefront.

**Fig. 2:**
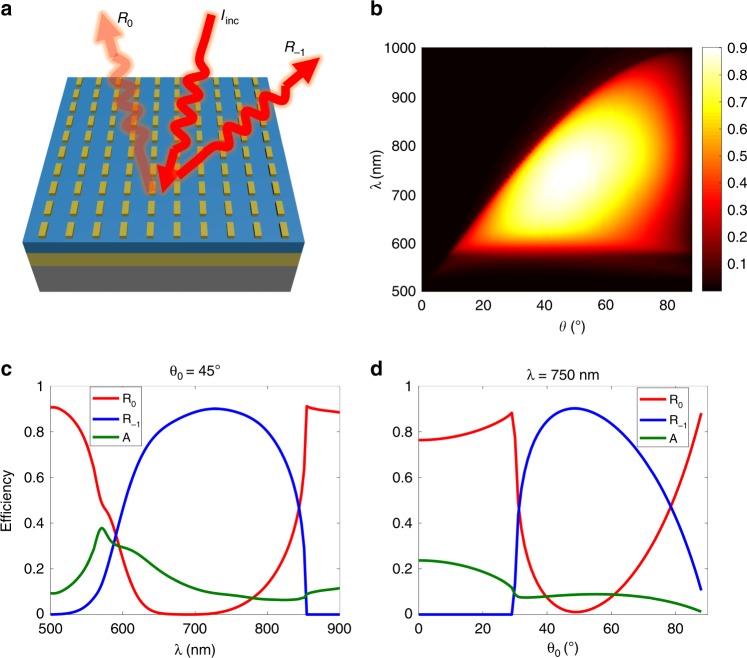
Diffraction properties of the EOD metagrating. **a** Schematic illustration of the periodic EOD metagrating and its diffraction channels. **b** The full-phase map of R_-1_ for varying incident angles from 0° to 90° and for wavelengths from 500 nm to 1000 nm for the given parameters of *L* = 200 nm, *w* = 90 nm, *h* = 130 nm, *p*_0_ = 500 nm, and *p*_y_ = 300 nm. **c**, **d** Diffraction spectra of the zeroth (R_0_) and -first (R_-1_) diffraction orders and absorption spectra (A) at a given incident angle of θ_0_ = 45^o^**c** and at a given wavelength of λ = 750 nm **d**, respectively

First, let us study the diffraction properties of the periodic metagrating, as shown in Fig. 2a. Basically, when the incidence condition satisfies Eqs. (1a), (1b) so that there are only the zeroth and −first diffraction order channels, the interplay between the localized plasmon resonance of the nanorod and the metallic background will give rise to the EOD. This can be analyzed by the coupled mode theory for a “one resonator (localized surface plasmon) and two decaying pathways (zeroth and −first diffraction orders)” system, as shown in Supplementary Fig. S2. Under the critical coupling condition (the coupling losses in the two decaying pathways are equal), the incident light can be totally funneled into the −first diffraction channel, while the zeroth diffraction can be completely suppressed (Supplementary Eq. (S8)). The critical coupling condition for the metagrating can be found via full-wave simulations of the metagrating by sweeping the geometrical parameters (length *L* and width *w*) of the plasmonic nanorod and the thickness, *h*, of the dielectric spacer. For the theoretical design and the following experimental realization, the metal was chosen to be silver^56^ and the dielectric spacer was silicon dioxide (*n* = 1.45).

The resonance condition for the EOD depends mainly on the length of the nanorod (Supplementary Figs. S3 and S4), while the bandwidth and peak value of the EOD can be optimized by the dielectric spacer thickness, *h* (Supplementary Fig. S5). When increasing *h*, the size of the nanorod should also be enlarged correspondingly to satisfy the critical coupling condition. To obtain a large bandwidth to reach the visible frequencies and a peak diffraction higher than 80%, we chose the optimized height of *h* = 130 nm (Supplementary Fig. S5a). At this optimized height, we also studied the influence of length and width of the nanorod on the diffraction efficiencies (Supplementary Fig. S5b and c). The high diffraction efficiencies are sustained for a wide range of nanorod lengths (120 nm–220 nm) and widths (70 nm–140 nm), which largely relaxes the fabrication demands. For the entire simulation process, the periodicity was fixed as p_x_ = p_0_ = 500 nm and p_y_ = 300 nm, so the visible and infrared wavelengths lie within the EOD regime, as dictated by Eqs. (1a) and (1b). Figure 2b shows the overall phase map of the diffraction efficiencies (R_−1_) as a function of incident angles and wavelengths for the structure with the selected parameters of *L* = 200 nm, *w* = 90 nm, and *h* = 130 nm. For practical implementation, the overlap between adjacent nanorods while constructing the detour phase hologram must be considered^48^; then, the practical phase modulation range in the experiment should be –π(p_0_-w)/p_0_ < φ < π(p_0_-w)/p_0_. Therefore, the selected nanorod width is slightly smaller than the optimized one (Supplementary Figs. S5c) to reduce the overlap ratio to be as low as 5% and to cover a phase modulation range that is large enough for a high-quality holographic image. R_−1_ reached a peak value as high as 90%, and the high diffraction efficiency ( > 50%) was sustained for a broad bandwidth from 600 nm to 850 nm (Fig. 2c) and for a wide range of angles from 30° to 70° (Fig. 2d). The upper bound of the diffraction efficiency was only limited by the Ohmic loss of the metal; a perfect unity diffraction efficiency is theoretically possible in the absence of metallic loss, as shown in Supplementary Fig. S6.

**Fig. 3:**
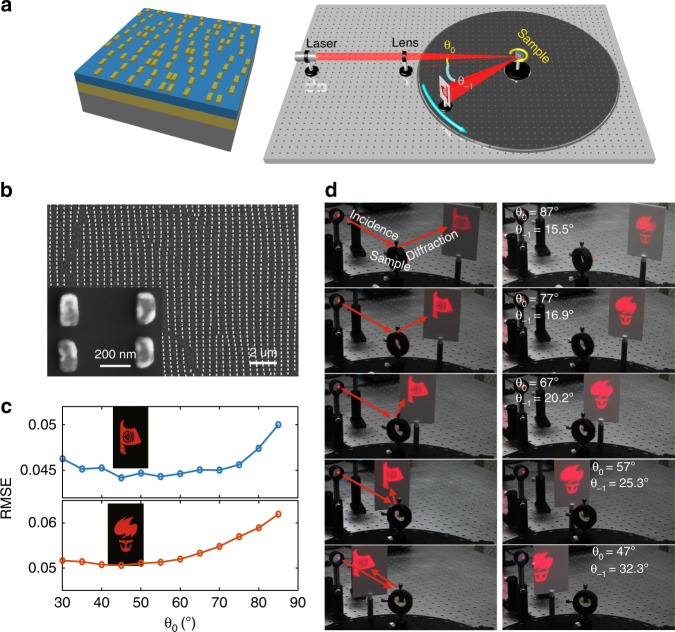
Performances of the metagrating holograms at different incident angles. **a** Illustration of the modulated metagrating hologram for arbitrary wavefront shaping (left) and the angle-resolved experimental setup for the holographic imaging (right). **b** SEM image of one of the designed metagrating samples modulated by the detour phase hologram. The inset depicts a zoomed-in view of the nanorods with uniform geometries of *L* = 200 nm, *w* = 90 nm, and *h* = 130 nm. **c** RMSE values and **d** photographs of reconstructed 'boat' (blue) and 'torch' (red) images at different incident angles

Based on the EOD metagrating, we designed two meta-holograms according to the dispersionless detour phase modulation rule. A schematic of the modulated metagrating structure is shown in Fig. 3a, which was readily fabricated using the electron beam lithography (EBL) technique, as shown in the scanning electron microscopy (SEM) image in Fig. 3b. The meta-atoms are uniform with an almost identical geometry (the same shape, size, and orientation), and only the position of those meta-atoms were modulated according to the phase profile of the pre-designed holographic image. The phase profiles of the designed meta-holograms were calculated using the Gerchberg–Saxton (GS) algorithm, with Fresnel diffraction formulas^57,58^, which produced 'boat' and 'torch' images, respectively, as shown in the insets of Fig. 3c. Figure 3d shows the experimentally reconstructed holographic images by illuminating the metagrating at different incident angles. Both the 'boat' and 'torch' images with high fidelities were reconstructed on a white screen. By rotating the meta-grating sample to vary the incident angle, holographic images with a negligible distortion could always be reconstructed in the −first diffraction direction (see Fig. 3d and Supplementary videos 1 and 2). Remarkably, the holographic images could even be reconstructed for a near-grazing incidence, as shown in Supplementary Fig. S7, which was out of the reach for previous phase-gradient meta-holograms.

**Fig. 4:**
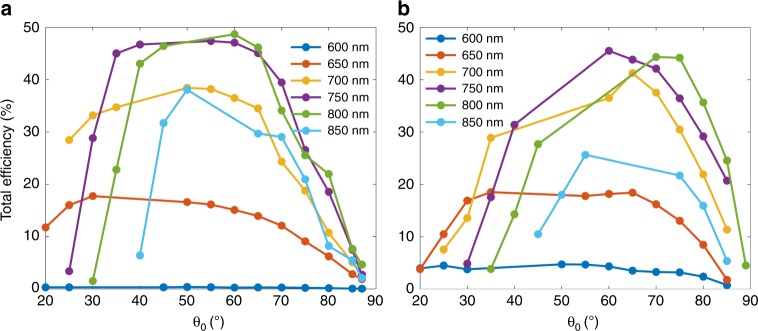
Measured diffraction efficiencies of the metagrating holograms. Diffraction efficiencies defined by the diffracted power over the total incident power at different incident angles and different wavelengths were experimentally measured for **a** a periodic metagrating and **b** a modulated metagrating hologram, respectively

To quantitatively determine the difference between the reconstructed holographic images and the target image, Fig. 3c shows the root-mean-square errors (RMSEs)^59^ defined  by  $RMSE = \sqrt {\mathop {\sum}\limits_{n = 1}^N {\mathop {\sum}\limits_{m = 1}^M {\left[ {I_{holo}\left( {x_n,y_m} \right) - I_{ideal}\left( {x_n,y_m} \right)} \right]^2/\left( {MN - 1} \right)} } }$ for different incident angles, where *I*_*holo*_ and *I*_*ideal*_ are the intensity distributions of the reconstructed and target images, respectively, and *N* and *M* are the pixel number in the *x* and *y* directions, respectively. As shown, the RMSEs sustain flat lines in the range of 30° to 70°, indicating that the reconstructed holographic images are indeed not distorted for a wide incident angle range. When the incident angle is larger than 70°, the RMSEs slowly increase, indicating only small distortions for extremely large incident angles. In addition to the high image quality, the measured overall efficiencies of these holographic images sustain a flat line at 18% from 30° to 70^o^ and gradually drop when the incident angle becomes smaller than 30° or larger than 70° (orange curve in Fig. 4b). The large angle tolerance of the proposed metagrating provides additional flexibility for meta-holograms, which is highly desirable for panoramic applications.

The experimentally measured diffraction efficiencies for both a periodic metagrating and a modulated metagrating hologram at different incident angles and wavelengths are shown in Fig. 4. For different incident wavelengths, the diffraction efficiency reaches a peak value at moderate incident angles of approximately 50°. At the central wavelength of approximately 750 nm, the diffraction efficiency is the highest. Both the angle position and spectral position of the efficiency peak for the periodic metagrating (Fig. 4a) are consistent with the theoretical results, as shown in Fig. 2c, d. The peak angle positions for the modulated metagrating (Fig. 4b) shift a little toward large angles because the modulated metagrating can be viewed as a grating with a spatially varying periodicity, which may affect both the peak angle and wavelength^47^. The maximum measured absolute efficiency for the metagrating is lower than the theoretical values. This difference can be explained by the following reasons. First, during the fabrication process, the titanium layer used for adhering the nanorods to the SiO_2_ layer significantly reduces the diffraction efficiency^12^. Second, the incident light spot inevitably exceeds the total sample area, especially for oblique incidence cases, which make the measured diffraction efficiency lower than the exact value. The obtained diffraction efficiencies here were for a single holographic image, which are much higher than for the previous binary meta-holograms in which twin images always exist^60–62^.

**Fig. 5:**
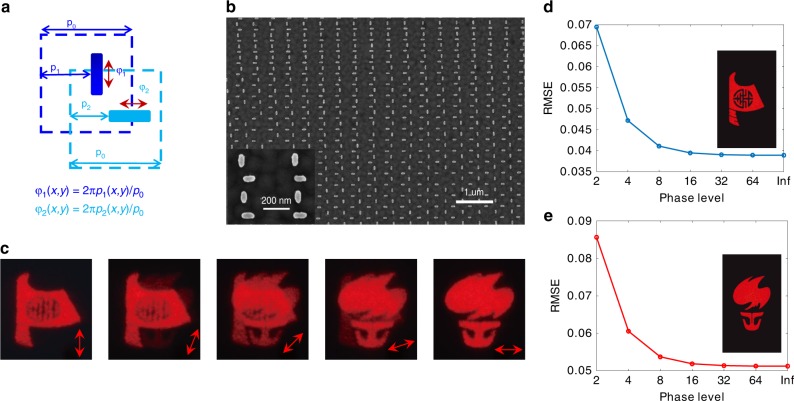
In-site polarization-multiplexed metagrating holograms. **a** Principle of the in situ polarization multiplexing by interleaving two sets of metagratings with vertically and horizontally aligned nanorods, respectively. The unit cells were spatially shifted by p_0_/2. Two phase profiles were independently encoded by the shifted displacements p_1_ and p_2_ for the nanorods. **b** SEM image of the fabricated polarization-multiplexed metagrating holograms. The inset depicts a zoomed-in view of the nanorods with uniform geometries of *L* = 130 nm, *w* = 50 nm, and *h* = 130 nm. **c** Experimentally captured holographic images by illuminating the metagrating hologram with different polarized incident light. **d**, **e** Comparison of the RMSE values of the multiplexed holographic images that were numerically reconstructed by metasurfaces based on different discrete phase levels

The EOD in the proposed metagrating is caused by the localized plasmon resonance of the anisotropic nanorod, which is preferentially excited when the polarization of the incident light is along the long axis of the nanorod. Leveraging this characteristic, we independently recorded two sets of holographic information by perpendicularly interleaving the aligned nanorod pair, as shown in Fig. 5a. The unit cell sizes of the two sets of metagrating holograms are both *p*_0_, whereas the relative position between the two unit cells is shifted by p_0_/2 to avoid near-field couplings. Two phase profiles, φ_1_(*x*, *y*) and φ_2_(*x*, *y*), can be independently encoded by displacements *p*_1_ and *p*_2_ of the two sets of nanorods with respect to their own unit cell boundary, respectively, to allow to distinctly reconstruct images in the same region at different incident light polarizations (in-site polarization multiplexing). For this purpose, we performed an optimization process to maximize the polarization extinction ratio for the diffracted light (Supplementary Fig. S8 and S9) to efficiently suppress the cross talk of the multiplexed images. Optical nanorod geometries of *L* = 130 nm and *w* = 50 nm were selected for the multiplexed metagrating holograms. Figure 5b shows the SEM image of the polarization-multiplexed metagrating hologram, in which the phase profiles of the 'boat' and 'torch' images are simultaneously encoded. By illuminating the metagrating with vertically polarized light, the 'boat' image appears (leftmost panel of Fig. 5c), which is determined by the phase profile φ_1_(*x*,*y*). When the polarization angles with respect to the vertical direction are 22.5°, 45°, and 67.5° (central three panels of Fig. 5c), both the 'boat' and 'torch' images appear corresponding to the superposition of the reconstructed images of φ_1_(*x*, *y*) and φ_2_(*x*, *y*), respectively. For horizontally polarized light (rightmost panel of Fig. 5c), the 'torch' image determined by φ_2_(*x*, *y*) distinctly appears. The distinct 'boat' and 'torch' images for the vertical and horizontal polarizations indicate the crosstalk-free performance of the multiplexed dual holographic images. Of note, our metagrating holograms modulate the phase profiles in a continuous manner to enable high-fidelity holographic images. In Fig. 5d, e, the RMSE plots numerically show that the quality of the multiplexed holographic image with continuous phase modulation significantly surpasses the quality of state-of-the-art metasurface holograms with discrete phase modulation levels.

## Discussion

In conclusion, we proposed a facile metagrating hologram approach to address the fundamental limits of both bandwidth and angle tolerance experienced by phase-gradient metasurfaces. Based on the EOD, we utilized uniformly shaped plasmonic nanorods above a dielectric spacer and a metallic background to tailor the diffraction channels to achieve near-unity diffraction efficiency. The in-plane displacement of individual nanorods in each unit cell was engineered for molding arbitrary wavefronts of light with an extreme angle tolerance in the broadband spectrum of 600 nm–850 nm. Furthermore, we experimentally demonstrated its application for high-fidelity holographic displays and in-site polarization multiplexing. Although we only demonstrated the metagrating holograms based on the reflection mode, the strategy can be readily applied to the transmission mode using all-dielectric metasurfaces. We envision that EOD metagrating holograms may have a wide impact on emerging new classes of flat optics with ultra-high performance and improved practicality.

## Materials and methods

### Simulation of diffraction efficiency and hologram design

The Finite element method (FEM) method implemented by COMSOL was used to simulate and optimize the metagrating parameters. The diffraction efficiencies of different orders (R_0_ and R_−1_) are calculated by setting incident plane waves with different incident angles. In the simulation, Floquet periodic boundary conditions are used for one unit cell of the metagrating. For the metagrating holograms design, the classical Gerchberg–Saxton algorithm with Fresnel diffraction formulas were applied to calculate the phase profile for the given images^57,58^.

### Fabrication and characterization of the metagrating sample

To fabricate the metagrating sample, a 130-nm silver film and a 130-nm SiO_2_ were successively deposited on a silicon substrate at first; and then the metagrating pattern was written above the SiO_2_ layer by the standard electron beam lithography; finally, 30 nm of silver film was deposited on top of the pattern and the final metagrating sample was formed by a lift-off process. For the sample characterization, a continuous wave laser at a wavelength of 633 nm was used to perform the holographic imaging, and a supercontinuum laser source was used to measure the diffraction efficiencies.

## Electronic supplementary material

Supplementary Information for Facile Metagrating Holograms with Broadband and Extreme Angle Tolerance

Supplementary Video 1

Supplementary Videos 2
