# Supplementary material for: Facile metagrating holograms with broadband and extreme angle tolerance
Source: Light Sci Appl. 2018 Oct 17;7:78. doi: 10.1038/s41377-018-0075-0 (PMC6193041; doi:10.1038/s41377-018-0075-0)
Supplement: Supplementary file 1 — Supplementary Information for Facile Metagrating Holograms with Broadband and Extreme Angle Tolerance [file 41377_2018_75_MOESM1_ESM.doc]

# Supplementary Information for

# Facile Metagrating Holograms with Broadband and Extreme Angle Tolerance

Zi-Lan Deng^1^,†, Junhong Deng^2,3^,†, Xin Zhuang^2^,†, Shuai Wang^1^, Tan Shi^1^, Guo Ping Wang^4^,*, Yao Wang^5^, Jian Xu^1^, Yaoyu Cao^1^, Xiaolei Wang^6^, Xing Cheng^2,^*, Guixin Li^2^,^3^,*, Xiangping Li^1^,*

^1^Guangdong Provincial Key Laboratory of Optical Fiber Sensing and Communications, Institute of Photonics Technology, Jinan University, Guangzhou 510632, China.

^2^Department of Materials Science and Engineering, Southern University of Science and Technology, 518055, Shenzhen, China.

^3^Shenzhen Institute for Quantum Science and Engineering, Southern University of Science and Technology, 518055, Shenzhen, China.

^4^College of Electronic Science and Technology, Shenzhen University, Shenzhen 518060, China.

^5^Materials Characterization and Preparation Center, Southern University of Science and Technology, 518055, Shenzhen, China.

^6^Institute of Modern Optics, Key Laboratory of Optical Information Science and Technology, Nankai University, Tianjin 300350, China.

^†^ These authors contributed equally to this work.

^*^E-mail: [gpwang@szu.edu.cn](mailto:gpwang@szu.edu.cn), [chengx@sustc.edu.cn](mailto:chengx@sustc.edu.cn), [ligx@sustc.edu.cn](mailto:ligx@sustc.edu.cn), xiangpingli@jnu.edu.cn

**Supplementary Fig. S1.** The comparison of (a) various typical phase-gradient metasurfaces by exploiting spatially-varying meta-atoms with gradient shapes, sizes or orientations, to mimic blazed gratings with flat profiles, and (b) Our proposed binary extraordinary optical diffraction (EOD) metagrating that can support high-efficiency beam steering without any geometry gradient. (c) The diffraction order chart in the *k*-space of the metagrating constructed by multiple wood’s anomaly (WA) lines, of which the green regions with only the 0^th^ and the (+,-)1^st^ diffraction orders denote the working regimes of the metagrating. The diffraction order chart is constructed by the multiple ordered WA lines determined by

*k*_x_±*m*2π/*p_0_*=±*k*_0_ (*m*=0, 1, 2, …), (S1)

where, *k_x_* is the parallel wavevector, *k_0_* is the overall wevevector, *p_0_* is the periodicity of the structure, and ±*m* is the diffraction order number. Above the ±*m*^th^ WA, the ±*m*^th^ propagating diffraction order begins to appear. Therefore, the working wavelength λ=2π/k_0_ and the incident angle *θ_0_*=asin(*k*_x_/*k*_0_) should be located in the momentum subspace surrounded by the 0^th^, 1^st^, -1^st^, and 2^nd^ (or -2^nd^) WA lines (Green patches). When the plasmonic gap resonance between the nanorod and the metal background is tailored in the green regime of the diffraction order chart, near-unity diffraction efficiency in the 1^st^ (or -1^st^) order can be obtained, with a complete suppression of the specular reflection ^1-3^.

**Supplementary Fig. S2.** Coupled mode theory for the resonance enhanced diffraction in systems with two decaying pathways: the 0^th^ diffraction pathway and the (±)1^st^ diffraction pathway. Assuming the complex amplitude of the resonator is *a*, the resonance frequency is ω_0_ and the total decaying rate is γ, the temporal evolution of the resonator amplitude can be written as ^4, 5^,

$\frac{da}{dt}=j\omega a=\left(j\omega_{0}-\gamma\right)a+\left(\begin{matrix} \kappa_{0} & \kappa_{1} \end{matrix}\right)\left(\begin{matrix} s_{0+} \\ s_{1+} \end{matrix}\right)$, (S2)

where, *κ_0_* and *κ_1_* are coupling constants between the resonance and the 0^th^ and -1^st^ diffraction channels, respectively; s_0+_ and s_1+_ are input complex amplitudes from the 0^th^ and -1^st^ diffraction channels into the resonator, respectively. On the other hand, light transporting among the 0^th^ and –1^st^ diffraction channels pass through both the direct pathway and the resonant pathway,

$\left(\begin{matrix} \text{s}_{0-} \\ s_{1-} \end{matrix}\right)=\mathbf{C}\left(\begin{matrix} \text{s}_{0+} \\ s_{1+} \end{matrix}\right)+\left(\begin{matrix} \kappa_{0} \\ \kappa_{1} \end{matrix}\right)a=\mathbf{S}\left(\begin{matrix} \text{s}_{0+} \\ s_{1+} \end{matrix}\right)$, (S3)

where, *s*_0-_ and *s*_1-_ are output complex amplitudes for the 0^th^ and -1^st^ diffraction channels, respectively; and

, (S4)

represents the scattering matrix for the direct pathway. **S** represents the overall scattering matrix for both the direct pathway and the resonant pathway. In addition, energy conservation and reciprocity give that

$\left(\begin{matrix} |\kappa_{0}|^{2} \\ |\kappa_{1}|^{2} \end{matrix}\right)=\left(\begin{matrix} 2\gamma_{0} \\ 2\gamma_{1} \end{matrix}\right)$, (S5)

$\text{ }\mathbf{C}\left(\begin{matrix} \kappa_{0} \\ \kappa_{1} \end{matrix}\right)^{*}=-\left(\begin{matrix} \kappa_{0} \\ \kappa_{1} \end{matrix}\right)$, (S6)

where, $\gamma_{0}$，$\gamma_{1}$ is the coupling rate from the resonator to the 0^th^ and 1^st^ diffraction pathways, and$\gamma_{0}+\gamma_{1}=\gamma$.

Combining Eqs. (S2)-(S6), we can obtain the total scattering matrix

$\mathbf{S}=\left(\begin{matrix} 1-\frac{2\gamma_{0}}{j\left(\omega-\omega_{0}\right)+\gamma} & \frac{-2\sqrt{\gamma_{0}\gamma_{1}}}{j\left(\omega-\omega_{0}\right)+\gamma} \\ \frac{-2\sqrt{\gamma_{0}\gamma_{1}}}{j\left(\omega-\omega_{0}\right)+\gamma} & 1-\frac{2\gamma_{1}}{j\left(\omega-\omega_{0}\right)+\gamma} \end{matrix}\right)$. (S7)

The reflection coefficient for the -1^st^ diffraction order is the *s*_1-_ due to *s*_0+_, namely,

$r_{1}=S_{21}=\frac{-2\sqrt{\gamma_{0}\gamma_{1}}}{j\left(\omega-\omega_{0}\right)+\gamma_{0}+\gamma_{1}}$. (S8)

It implies that, when $\gamma_{0}=\gamma_{1}$, $r_{1}$ can reach unity at the resonance frequency *ω_0_*.

**Supplementary Fig. S3.** The simulated diffraction efficiency spectra of the (a) 0^th^ (R_0_), and (b) -1^st^ (R_-1_) diffraction order of the periodic metagrating for varying the length of the nanorod when the thickness of the dielectric spacer is *h*=50 nm. The nanorod width and the unit cell size are fixed as *w*=50 nm, p_0_=500 nm, p_y_=300 nm, respectively. The plasmon resonance causes the dip of R_0_ and the peak of R_-1_, the resonance wavelength is linearly dependent on the length of the nanorod. Due to the strong absorption loss of the plasmonic resonance, the peak value of R_-1_ only reaches 0.4.

**Supplementary Fig. S4.** The simulated diffraction efficiency spectra of the (a) 0^th^ (R_0_), and (b) -1^st^ (R_-1_) diffraction order of the periodic metagrating for varying the length of the nanorod when the thickness of the dielectric spacer is *h*=130 nm. The nanorod width and unit cell size are fixed as w=50 nm, p_0_=500 nm, p_y_=300 nm, respectively. Both the bandwidth and peak value of R_-1_ peak increase compared with the case of *h*=50 nm, because the plasmonic resonance becomes weak, and absorption loss is largely reduced.

**Supplementary Fig. S5.** Diffraction efficiency spectra (R_-1_) of the EOD metagrating for different (a) dielectric spacer thicknesses *h*, (b) nanorod lengths *L*, and (c) nanorod widths *w*, respectively, at the incident angle 45^o^. The unit cell size for all cases is fixed as p_0_=500 nm, p_y_=300 nm, respectively.

**Supplementary Fig. S6.** Perfect unitary diffraction efficiency of the metagrating when the Ohmic loss of metal is out of consideration. (a) The parameters of the metagrating are w=50 nm, L=100 nm, h=50 nm, p_0_=500nm, p_y_=300nm, respectively, which shows a narrow banded EOD with perfect unity diffraction at the wavelength of λ=720 nm. (b) The parameters of the metagrating are chosen as w=90 nm, L=200 nm, h=130 nm, p_0_=500nm, p_y_=300nm, respectively, which shows a broadband EOD from 650 nm to 800 nm with diffraction efficiency exceeding 90%, and the perfect unitary diffraction occurs at λ=700 nm.

**Supplementary Fig. S7.** The photograph of the experimental setup from the top view for the case of an extremely large incident angle (87.5^o^) that is close to the grazing incidence. The holographic image persists in the diffraction direction with negligible distortion as captured by a white screen.

**Supplementary Fig. S8.** Optimization of the polarization extinction ratio for the metagrating composed of vertically aligned nanorods at the EOD central wavelength of 750 nm. (a) The diffraction efficiency of TE incident light for varying nanorod width *w_x_* and *w_y_,* in the *x* and *y* direction, respectively. As the *w_x_* increases, the range of *w_y_* for a high diffraction efficiency R_TE-1_ increases. (b) The diffraction efficiency of TM incident light for varying *w_x_* and *w_y_*, the diffraction efficiency R_TM-1_ increases with the *w_x_*. (c) The extinction ratio (ER) defined as R_TE-1_/ R_TM-1_, for varying *w_x_* and *w_y_*. The maximum ER 180 occurs at *w_x_=50 nm* and *w_y_**=130 nm.*

**Supplementary Fig. S9.** Optimization of the polarization extinction ratio for the metagrating composed of horizontally aligned nanorods at the EOD central wavelength of 750 nm. (a) The diffraction efficiency of TE incident light for varying nanorod width *w_x_* and *w_y_,* in the *x* and *y* direction, respectively. The diffraction efficiency R_TE-1_ increases with the *w_y_*. (b) The diffraction efficiency of TM incident light for varying *w_x_* and *w_y_*, the diffraction efficiency R_TM-1_ increases with the *w_x_*. (c) The extinction ratio (ER) defined as R_TM-1_/ R_TE-1_, for varying *w_x_* and *w_y_*. The maximum ER 20 occurs at *w_x_=130 nm* and *w_y_=50 nm.*

**References**

1 Deng Z-L, Zhang S, Wang GP. A facile grating approach towards broadband, wide-angle and high-efficiency holographic metasurfaces. *Nanoscale* 2016; **8**: 1588-1594.

2 Deng Z-L, Zhang S, Wang GP. Wide-angled off-axis achromatic metasurfaces for visible light. *Opt Express* 2016; **24**: 23118-23128.

3 Ra’di Y, Sounas DL, Alù A. Metagratings: Beyond the Limits of Graded Metasurfaces for Wave Front Control. *Phys Rev Lett* 2017; **119**: 067404.

4 Haus HA. *Waves and Fields in Optoelectronics*. (Prentice Hall, 1983).

5 Wang KX, Yu Z, Sandhu S, Liu V, Fan S. Condition for perfect antireflection by optical resonance at material interface. *Optica* 2014; **1**: 388-395.
