# Supplementary figures and images for: Facile metagrating holograms with broadband and extreme angle tolerance
Source: Light Sci Appl. 2018 Oct 17;7:78. doi: 10.1038/s41377-018-0075-0 (PMC6193041; doi:10.1038/s41377-018-0075-0)

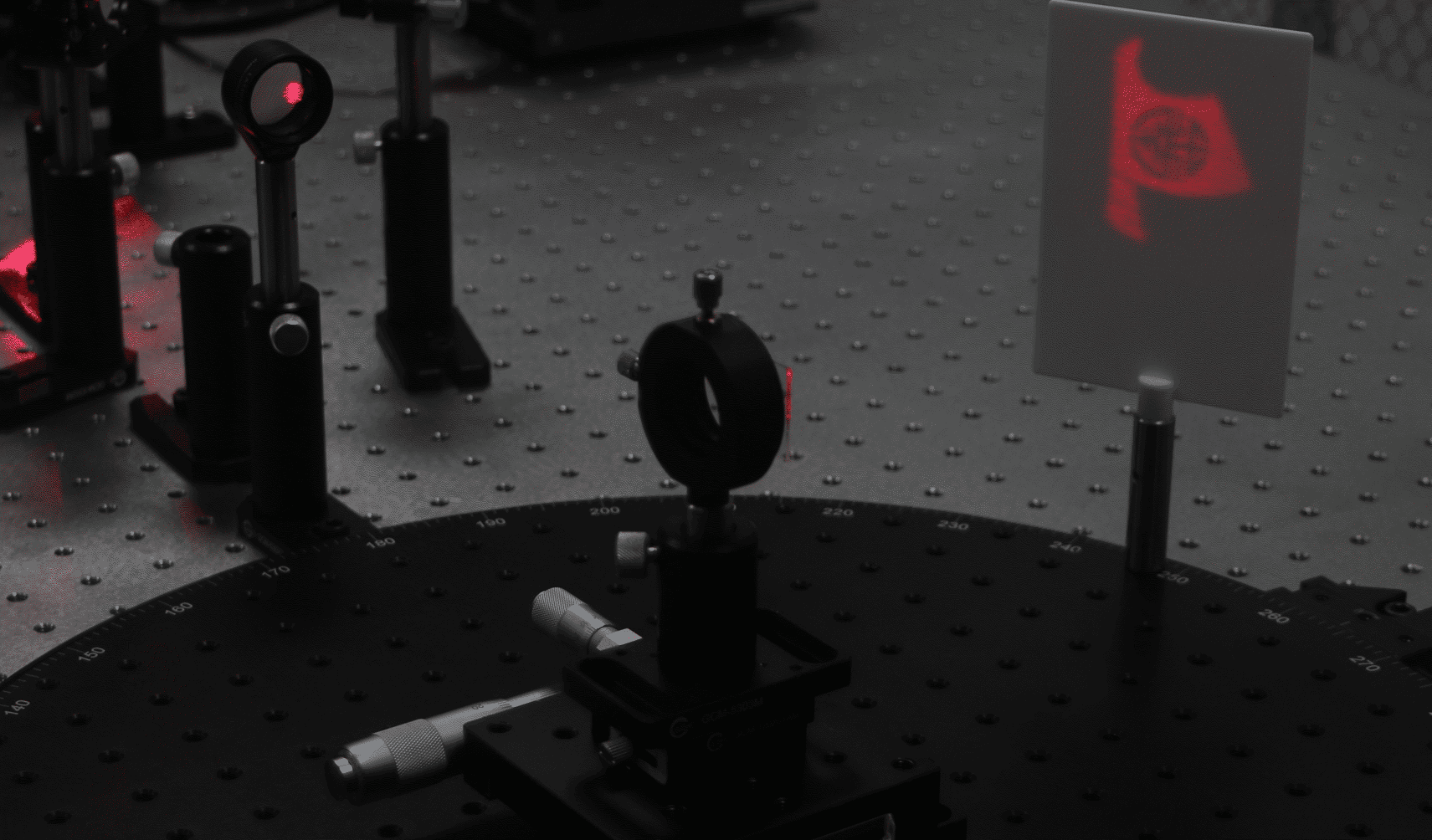

Supplement: Supplementary file 2 — Supplementary Video 1 [file 41377_2018_75_MOESM2_ESM.gif]

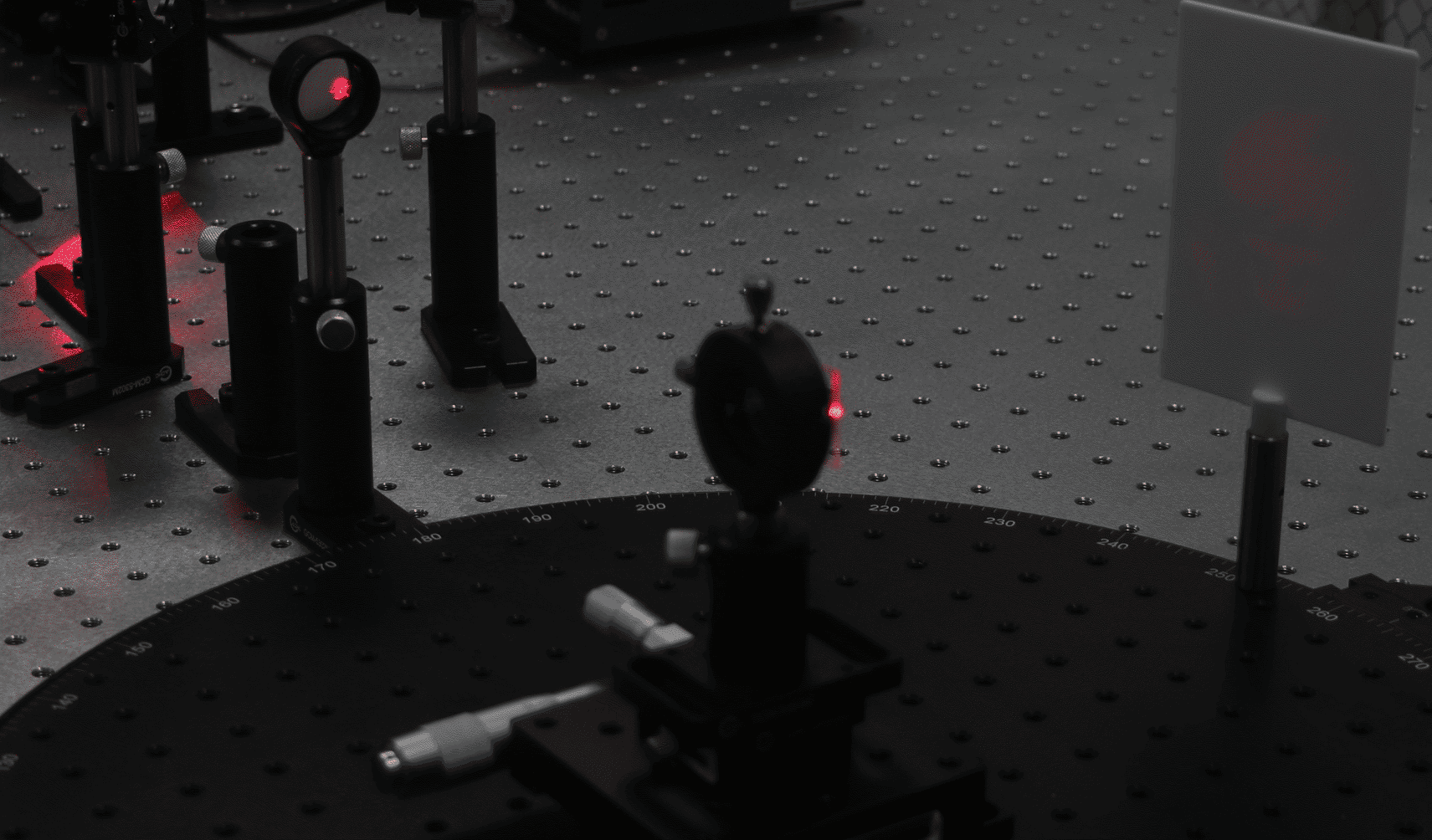

Supplement: Supplementary file 3 — Supplementary Videos 2 [file 41377_2018_75_MOESM3_ESM.gif]
